# Antimicrobial Activity of *Rhoeo discolor* Phenolic Rich Extracts Determined by Flow Cytometry

**DOI:** 10.3390/molecules201018685

**Published:** 2015-10-14

**Authors:** Rebeca García-Varela, Rebeca M. García-García, Bertha A. Barba-Dávila, Oscar R. Fajardo-Ramírez, Sergio O. Serna-Saldívar, Guy A. Cardineau

**Affiliations:** 1 Centro de Biotecnología FEMSA, Tecnologico de Monterrey, Campus Monterrey, Ave Eugenio Garza Sada 2501, Monterrey 64849, N.L., Mexico; E-Mails: rbk_varela@hotmail.com (R.G.-V.); rebeca.garcia.garcia@itesm.mx (R.M.G.-G.); bertha.barba@itesm.mx (B.A.B.-D.); sserna@itesm.mx (S.O.S.-S.); 2 Centro de Agrobiotecnología, Tecnologico de Monterrey, Campus Monterrey, Ave Eugenio Garza Sada 2501, Monterrey 64849, N.L., Mexico; 3 Centro de Innovación y Transferida en Salud, Escuela de Medicina, Tecnologico de Monterrey, Campus Monterrey, Ave Morones Prieto No. 3000 Pte., Col. Los Doctores, C.P. Monterrey 64710, N.L., Mexico; E-Mail: ofajardo@itesm.mx

**Keywords:** antifungal, antimicrobial, *Candida albicans*, *Escherichia coli*, flow cytometry, *Listeria innocua*, phenolic compounds, *Pseudomonas aeruginosa*, *Rhoeo discolor*, *Streptococcus mutans*

## Abstract

Traditional medicine has led to the discovery of important active substances used in several health-related areas. Phytochemicals in *Rhoeo discolor* extracts have proven to have important antimicrobial activity. In the present study, our group determined the antimicrobial effects of extracts of *Rhoeo discolor*, a plant commonly used in Mexico for both medicinal and ornamental purposes. We evaluated the *in vitro* activity of phenolic rich extracts against specifically chosen microorganisms of human health importance by measuring their susceptibility via agar-disc diffusion assay and flow cytometry: Gram-positive *Listeria innocua* and *Streptococcus mutans*, Gram-negative *Escherichia coli* and *Pseudomonas aeruginosa*, and lastly a fungal pathogen *Candida albicans*. Ten different extracts were tested in eight different doses on all the microorganisms. Analytical data revealed a high content of phenolic compounds. Both agar-disc diffusion assay and flow cytometry results demonstrated that *Pseudomonas aeruginosa* was the least affected by extract exposure. However, low doses of these extracts (predominantly polar), in a range from 1 to 4 μg/mL, did produce a statistically significant bacteriostatic and bactericidal effect on the rest of the microorganisms. These results suggest the addition of certain natural extracts from *Rhoeo discolor* could act as antibacterial and antimycotic drugs or additives for foods and cosmetics.

## 1. Introduction

Since the beginning of medicinal research, a primary focus has been directed towards identification of natural resources and materials [1] with the potential to treat and/or cure sometimes complex diseases or their symptoms, resulting in the development of traditional medicine [2]. This folk knowledge, based predominantly on theories and indigenous experience to maintain good health and disease prevention, has provided important information regarding medicinal plants and practices [3]. The study of these traditions has led to the identification of plants worth researching for therapeutic potential, and the discovery of important active substances [1] such as coumarin, extracted from *Melilotus officinalis*, known for its antimicrobial activity [4]. Traditional medicine is based on uses, practices, and recipes for the consumption or topical application of these plants, rudimentary extracts or plant tissue. The possibility of obtaining health benefits by adding certain foods in the regular diet derived from plants with identified medicinal or therapeutic value may offer improved life and health to the consumer [5,6].

In Mexico, traditional medicine is widely used in parallel with modern medicine. *Rhoeo discolor* (*R. discolor*), whose modern taxonomical accepted name is *Tradescantia spathacea*, and also commonly known as “purple maguey”, has its origin in the tropics of the Gulf of Mexico, the Caribbean and the coasts of Central America. Today, it has spread to many other regions around the world. The leaves have been used in regional native cultures, consumed mostly in infusions or in direct skin contact, to treat allergic rhinitis, superficial mycosis, ulcers, as a broad-spectrum anti-inflammatory and dermatological agent, and also as a treatment for cancer [2]. These properties have been attributed to the content of bioactive molecules such as anthocyanins [7]. The effectiveness of traditional treatments with *R. discolor* has been shared among communities, but it has never been subjected to a systematic scientific scrutiny; although it has proven to be antigenotoxic, antimutagenic and antioxidant, further scientific information about this plant is necessary to ensure side effects do not overcome benefits [8].

Essential analyses of *R. discolor*, such as a broad evaluation of potential antimicrobial activity, have yet to be exploited. To address this, we report the effect of 10 different extracts of *R. discolor* on five selected microorganisms: two Gram-negative bacteria, (a) *Escherichia coli* (*E. coli*), the most studied prokaryotic organism commonly found in human and other animal intestines, frequently associated with food borne illness [9], and (b) *Pseudomonas aeruginosa* (*P. aeruginosa*), responsible for many respiratory tract infections and the main cause of mortality amongst cystic fibrosis patients [10]; two Gram-positive bacteria, (c) *Listeria innocua* (*L. innocua*), a non-pathogenic strain model for *Listeria monocytogenes* (*L. monocytogenes*), which causes listeriosis, an infection of the central nervous system, often related to food poisoning, with a high mortality rate [11,12], and (d) *Streptococcus mutans* (*S. mutans*), the chief etiologic agent responsible for cavities and dental plaque [13]; and, finally, (e) *Candida albicans* (*C. albicans*), a yeast responsible for infections in mucosal tissue such as the gastrointestinal tract, oral cavity and more commonly vaginal infections [14]. Treatment of the diseases resulting from infection by these microorganisms could be aided by a potentially bioactive extract that may not only serve as a therapeutic but as a preventive safeguard.

Members of the *Commelinaceae* family, including *R. discolor*, have been previously explored as sources of antioxidants and antimicrobials [15,16] However, the direct effect of the extracts on a variety of microorganisms of importance to human health has yet to be explored.

## 2. Results and Discussion

### 2.1. Agar Diffusion Assays

**Figure 1:**
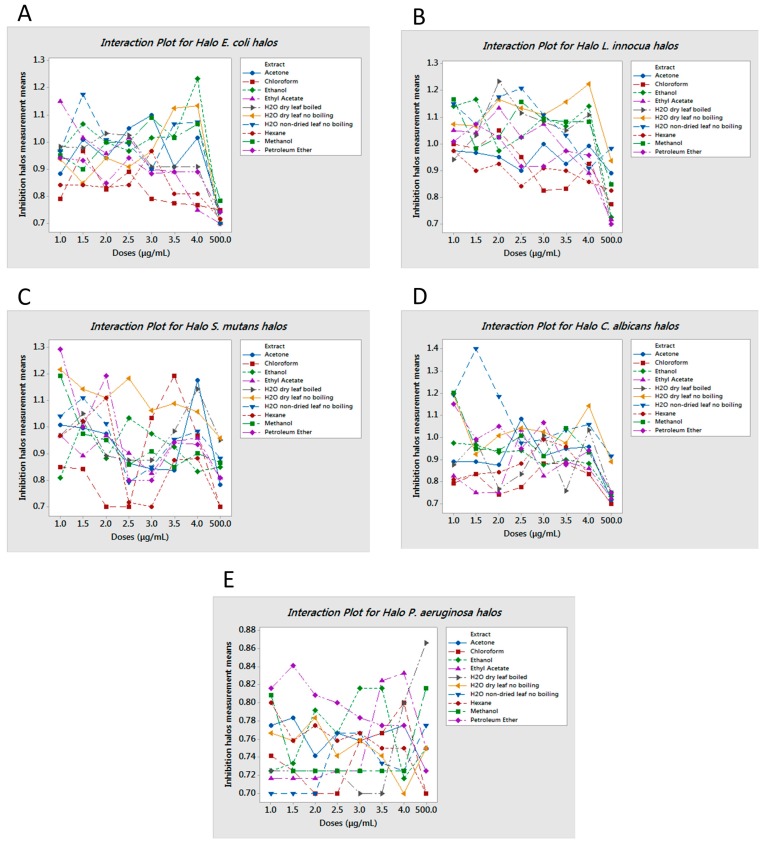
Interaction halo plots, (**A**) *E. coli*; (**B**) *L. innocua*; (**C**) *S. mutans*; (**D**) *C. albicans*; and (**E**) *P. aeruginosa*. Paper discs were 0.7 cm in diameter. Halo diameter in cm is given in the Y-axis *vs.* dose concentration in µg/mL in the X-axis. Inhibition was measured based on halo diameter in excess of 0.7 cm with higher peaks indicating the most active doses of extracts that produce inhibition halos of greater diameter.

**Figure 2:**
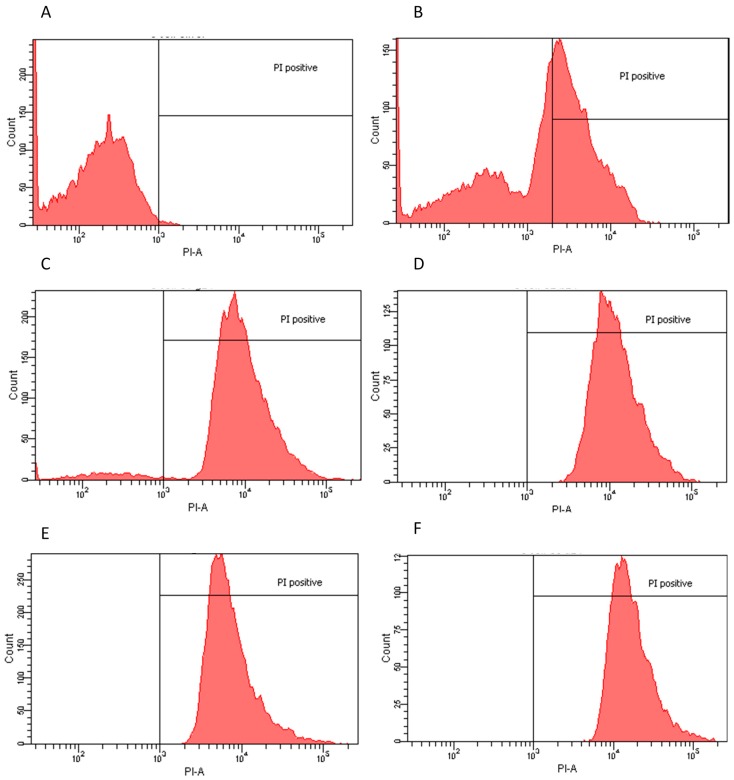
Flow cytometry histograms derived from scatter plots showing fluorescence of live (<103) or dead (>103) *E.* *coli* stained with propidium iodine (PI) post 24 h incubation with controls or extracts. X-axis corresponds to the PI positive marked microorganisms and Y-axis to the total cell count. (**A**) *E.* *coli* sample PI negative control, 96.7% viability; (**B**) *E.* *coli* incubated for 30 min with 70% ethanol PI positive control, 48% viability; (**C**) water dry leaf non-boiled extract 4 μg/mL, 5.7% viability; (**D**) water non-dried leaves non-boiled, 1.5 μg/mL 0.1% viability; (**E**) ethanol 4 μg/mL, 0.1% viability; and (**F**) ethyl Acetate 1 μg/mL, 0.3% viability.

**Figure 3:**
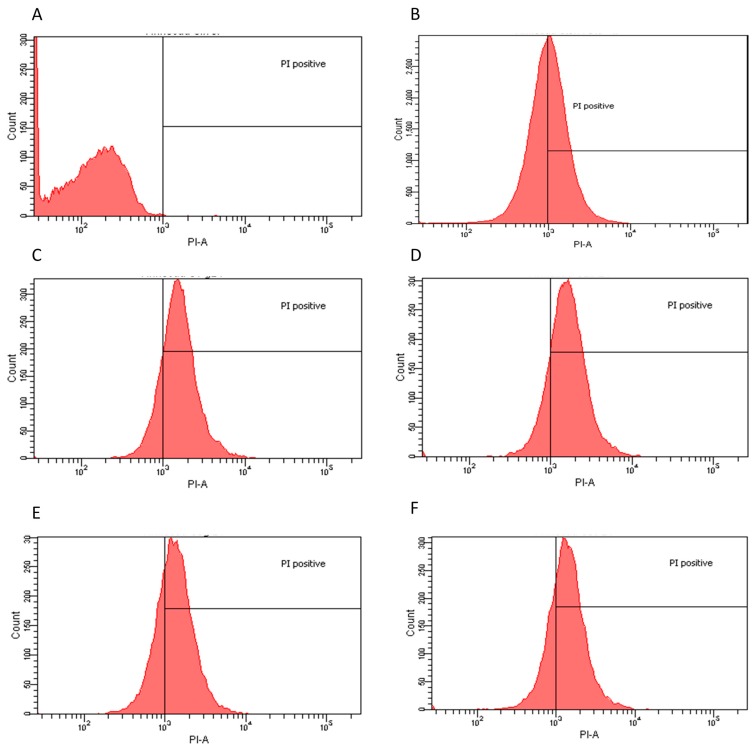
Flow cytometry histograms derived from scatter plots showing fluorescence of live (<103) or dead (>103) of *L. innocua* stained with PI post 24 h incubation with controls or extracts. X-axis corresponds to the PI positive marked microorganisms and Y-axis to the total cell count. (**A**) *L. innocua* sample PI negative control, 99.9% viability; (**B**) *L. innocua* incubated for 30 min with ethanol 70% PI positive control 46.4% viability; (**C**) water dry leaf non-boiled extract 4 μg/mL, 17.5% viability; (**D**) water non-dried leaves non-boiled 2.5 μg/mL, 16.7% viability; (**E**) ethanol 4 μg/mL, 28.5% viability; and (**F**) water dry leaf boiled 2 μg/mL, 25.6% viability.

**Figure 4:**
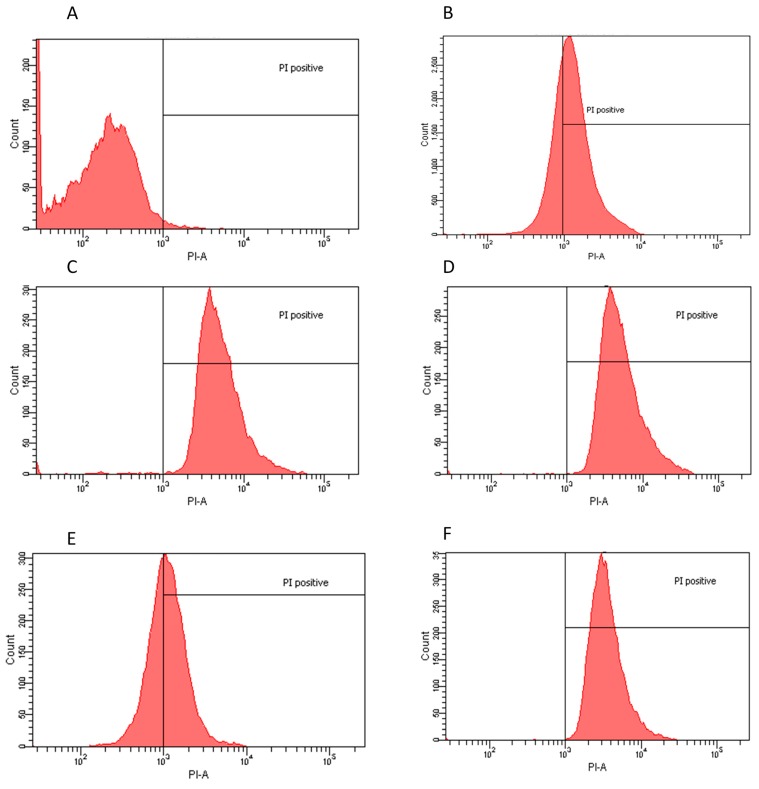
Flow cytometry histograms derived from scatter plots showing fluorescence of live (<103) or dead (>103) of *S.* *mutans* stained with PI post 24 h incubation with controls or extracts. X-axis corresponds to the PI positive marked microorganisms and Y-axis to the total cell count. (**A**) *S.* *mutans* sample PI negative control, 99.8% viability; (**B**) *S.* *mutans* incubated for 30 min with ethanol 70% PI positive control 30.6% viability; (**C**) water dry leaf non-boiled extract 1 μg/mL, 1.5% viability; (**D**) Acetone 4 μg/mL, 0.5% viability; and (**E**) Petroleum ether 1 μg/mL, 41.6% viability; (**F**) Chloroform 3.5 μg/mL, 0.3% viability.

**Figure 5:**
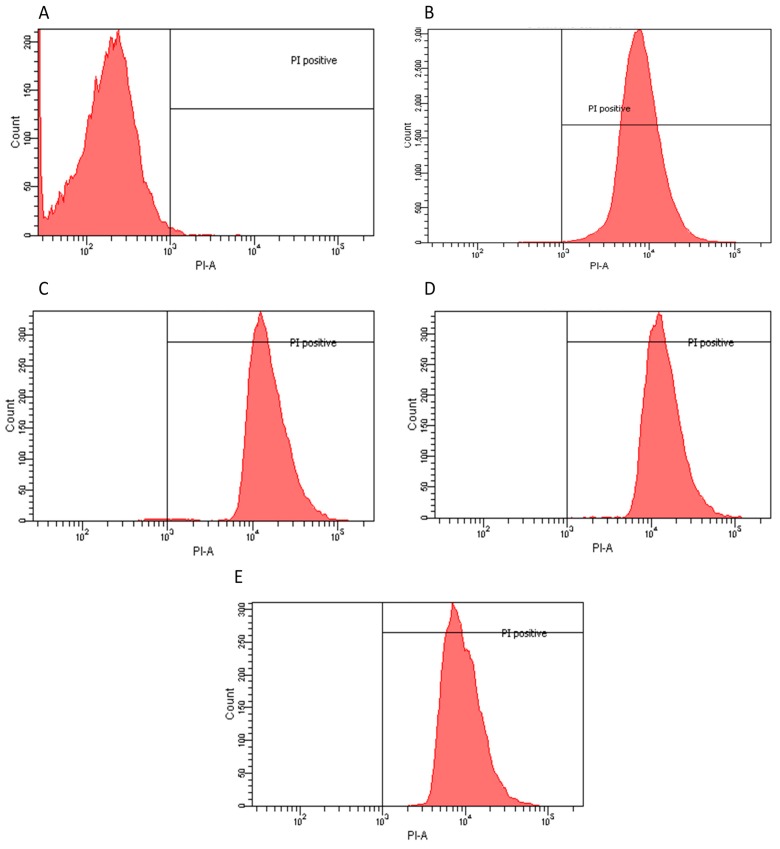
Flow cytometry histograms derived from scatter plots showing fluorescence of live (<103) or dead (>103) of *C.* *albicans* stained with PI post 24 h incubation with controls or extracts. X-axis corresponds to the PI positive marked microorganisms and Y-axis to the total cell count. (**A**) *C. albicans* sample PI negative control, 99.8% viability; (**B**) *C.* *albicans* incubated for 30 min with ethanol 70% PI positive control 0.1% viability; (**C**) water dry leaf non-boiled extract 1 μg/mL, 0.6% viability; (**D**) methanol 1 μg/mL, 0.1% viability; and (**E**) water non-dried leaves non-boiled 1.5 μg/m, 0.2% viability.

The inhibitory effect of some of the crude extracts was supported by statistical data. We considered extracts to be bioactive if a clear halo was produced at least 3 mm wider than the agar diffusion assay disc. Based on the obtained results, we found that not all extracts or all the doses were effective on all the microorganisms studied herein (Figure S1 in supplementary materials). However, it is important to note that better results were obtained by the extracts formulated using more polar solvents and/or water (Table S1). As depicted in Figure 1 (panels A, B, and D, respectively), *E. coli*, *L. innocua* and *C. albicans* were readily responsive to several treatments, specifically those in which the extraction was performed with polar solvents or water. In contrast, better results were obtained with *S. mutans* when exposed to non-polar solvent extracts that were dosed at low concentrations, from 1 to 4 μg/mL, as shown in Figure 1C. Regardless, a significant response to the determined aqueous extracts was also achieved [17]. In the case of *P. aeruginosa*, none of the extract-dose interactions showed a significant growth inhibition with *p*-value < 0.05 (Figure 1E). Results were reproducible with minimal error, as shown in Figure S2. However, only the most effective extracts and doses were analyzed in more detail and submitted to flow cytometry testing. The extract–dose analyzed by flow cytometry were: for *E. coli*, ethanol 4 μg/mL, water non-dried leaf non-boiled 1.5 μg/mL, ethyl acetate 1 μg/mL and water dry leaf non-boiled 4 μg/mL (Figure 2); for *L. innocua*, water dry leaf non-boiled 4 μg/mL, water dry leaf boiled 2 μg/mL, ethanol 4 μg/mL and water non-dried leaf non-boiled 2.5 μg/mL (Figure 3); *S. mutans*, petroleum ether 1 μg/mL, chloroform 3.5 μg/mL, acetone 4 μg/mL and water dry leaf non-boiled 1 μg/mL (Figure 4); *C. albicans*, water non-dry leaf non-boiled 1.5 μg/mL, methanol 1 μg/mL and water dry leaf non-boiled 1 μg/mL (Figure 5) *P. aeruginosa* was not submitted to further analysis due to lack of response to the extracts.

### 2.2. Flow Cytometry Assay

Flow Cytometry confirmed the agar diffusion assay results, demonstrating that the most efficient extracts, as determined by halo diameter, have a bactericidal/fungicidal or bacteriostatic effect. Extract-dose bactericidal effect was determined by a 98% growth inhibition [18]. *E. coli* and *C. albicans* had the highest population reduction rates, suggesting both a bactericidal and fungicidal effect since the agar-disc diffusion assays produced clear halos and confirming that the water and polar solvent extracts were the most efficient in these cases. *S. mutans* also responded favorably to a water extract with a 98.5% population reduction. Chloroform and acetone extracts also eliminated over 99% of the population. These three extracts could be considered bactericides and/or fungicides. However, petroleum ether extract had a lower efficiency, suggesting only a possible bacteriostatic effect since there was also a clear halo in the agar assays. Moreover, *L. innocua*, when exposed to water and polar solvent extracts, showed a clear inhibition in the agar-disc diffusion assays, whereas lower population decreases were observed in flow cytometry compared to the other microorganisms, suggesting a parallel bacteriostatic-bactericidal effect (Table 1). This effect is commonly known as the “Phoenix” effect [19], which results from the formation of pores in the membrane caused by the extracts. As long as the microorganisms are in the presence of such compounds their growth is inhibited; however, they are still alive. It is important to note that even though the extracts required more time to cause an effect, 24 h, the results in population growth inhibition were higher than those obtained by exposure to 70% ethanol, commonly used as a standard disinfecting procedure.

**Table 1:** Flow Cytometry Analysis.

Extract	Dose	Effect	PI Positive
*E. coli*			
Ethanol	4 μg/mL	Bactericidal	99.90%
Water non-dried leaf non-boiled	1.5 μg/mL	Bactericidal	99.90%
Ethyl Acetate	1 μg/mL	Bactericidal	99.70%
Water dry leaf non-boiled	4 μg/mL	Bactericidal	94.30%
*L. innocua*			
Water dry leaf non-boiled	4 μg/mL	Bacteriostatic	82.50%
Water dry leaf boiled	2 μg/mL	Bacteriostatic	74.40%
Water non-dried leaf non-boiled	2.5 μg/mL	Bacteriostatic	83.30%
Ethanol	4 μg/mL	Bacteriostatic	71.50%
*S. mutans*			
Petroleum Ether	1 μg/mL	Bacteriostatic	58.40%
Chloroform	3.5 μg/mL	Bactericidal	99.70%
Acetone	4 μg/mL	Bactericidal	99.50%
Water dry leaf non-boiled	1 μg/mL	Bactericidal	98.50%
*C. albicans*			
Water non-dry leaf non-boiled	1.5 μg/mL	Fungicide	99.80%
Methanol	1 μg/mL	Fungicide	99.90%
Water dry leaf non-boiled	1 μg/mL	Fungicide	99.40%

Based on the results of the Agar Diffusion Assay, flow cytometry was used to determine the bacteriostatic or bactericidal effects of specific extracts against selected pathogens. Population reduction was determined by PI positive cells (PI-A), represented on the right side of the 10^3^ PI-A mark in the charts, via comparison between a control sample of each population (no extract treatment) and test samples of each population (extract treatment).

### 2.3. Total Phenolic Count

The presence of phenolic compounds was elucidated first by the Folin-Ciocalteu’s assay and reported absorbance values were converted in concentration data when analyzed against a Gallic acid curve (Table 2).

**Table 2:** Total phenolic count in *R. discolor* extracts by Gallic acid curve.

Extract	µg GAE/mg
Extract #1: H_2_O dry leaf, non-boiled	8.5 ± 3.7
Extract #2: H_2_O non-dried leaf, non-boiled	7.5 ± 1.5
Extract #3: H_2_O dry leaf, boiled	16.9 ± 3.7
Extract #4: Methanol (MeOH)	1.5 ± 0.7
Extract #5: Ethanol (EtOH)	1.6 ± 0.2
Extract #6: Ethyl Acetate (EtAc)	9.4 ± 8.8
Extract #7: Acetone (Ac)	5.5 ± 1.1
Extract #8: Petroleum Ether (PtEt)	1.4 ± 2.4
Extract #9: Chloroform (CHCl_3_)	1.7 ± 2.5
Extract #10: Hexane (Hx)	0.7 ± 0.0

Total phenolic count was determined by using a Gallic acid standard curve. Data is expressed as µg of Gallic acid equivalent per 100 mg of sample (µg GAE/100 mg) and given as the Mean ± SD.

### 2.4. Analytical Data

**Figure 6:**
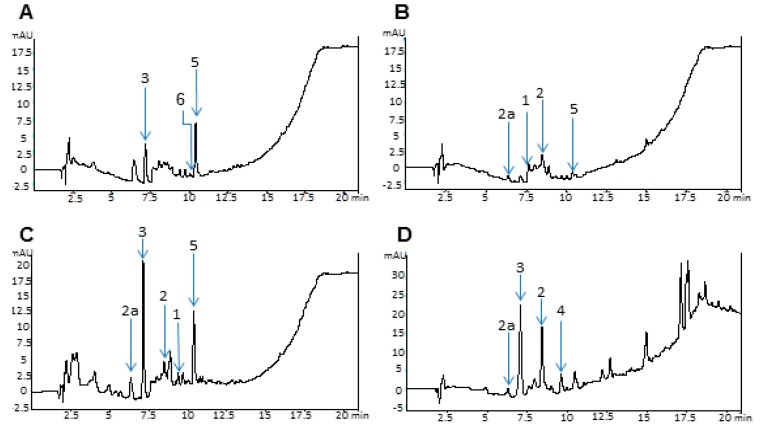

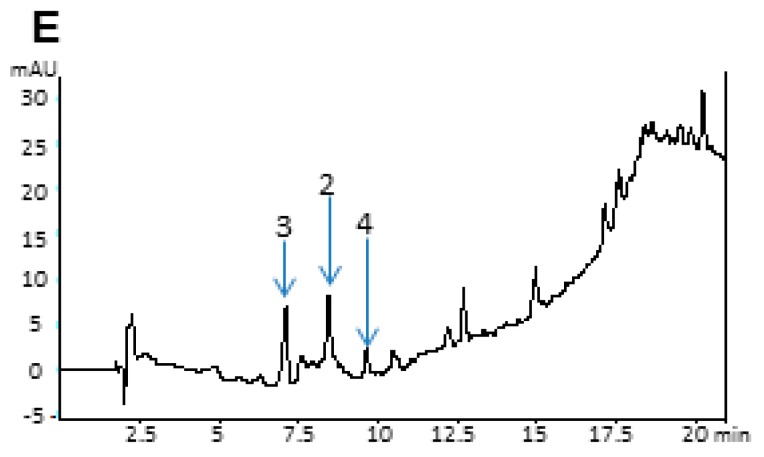
High-Performance liquid chromatography (HPLC) chromatogram at 254 nm of *R. discolor* extracts: (**A**) Water dry leaves non-boiled extract; (**B**) Water non-dry leaves non-boiled extract; (**C**) Water dry leaves boiled extract; (**D**) Acetone extract; (**E**) Ethyl Acetate extract. Selected chromatographic peaks correspond to the following: (**1**) Ferulic acid, (**2**) Vanillic acid, (**2a**) Glycosylated vanillic acid, (**3**) Chlorogenic acid, (**4**) P-coumaric, (**5**) Rhoeonin, and (**6**) Anthocyanin pigment No. 2.

Having demonstrated an important antimicrobial and antifungal effect, analytical protocols were performed to determine the composition of the different extracts. The resulting data revealed an elevated content of phenolic compounds such as terpenes, saponins, coumaric acid, ferulic acid and mostly anthocyanins, amongst others not yet identified (Figure 6). Nevertheless, further analysis is needed to determine the exact extract composition (experiments in process).

Plant secondary metabolites have been known to have a defensive role against microbes and to protect the plant while under stress [20]. *R. discolor* showed a high content of these metabolites, such as flavonoids, saponins, carotenoids, anthocyanins, terpenoids, ferulic acid, chlorogenic acid, vanillic acid and also *p*-coumaric acid and steroidal compounds, although most of the phytocomposition still remains unclear and undescribed [1]. Nevertheless, most of the compounds have been reported as beneficial in several cases [21], especially as antimicrobial agents [22]. Analytical data confirmed an elevated content of anthocyanins such as rhoeonin [16,23] and pigment No. 2 that corresponds to cyanidin 3-arabinosylglucoside-7,3ʹ-diglucoside with three molecules of caffeic acid [23]. This family of compounds has been previously tested to determine their utility as antimicrobial agents [24]. Ferulic, vanillic, chlorogenic and *p*-coumaric acid were also detected in the chromatogram and then identified by their individual spectra [25]; both the latter two acids have demonstrated an antimicrobial effect against some Gram Positive and Gram Negative bacteria [26,27,28]. On the other hand, evidence of small quantities of terpenes, such as saponins, was also found in the chromatographs and spectra; it is also documented that these phytocompounds have important antimicrobial activity [29]. It is well known that optimal flavonoid extraction methods require polar solvents, and even water, to obtain the majority of these compounds [30], which supports the higher effectiveness of the more polar extractions. Due to the known capacity of flavonoids to inhibit spore germination of plant fungal pathogens, it has been suggested that the same use may apply against human fungal pathogens [31]. In the present study we demonstrated the high growth inhibition effect in *C. albicans* when exposed to *R. discolor* polar extracts, reinforcing the hypothesis of a possible action mechanism in the case of Fungi. These extracts appear to be more effective in *C. albicans* due to the different composition of the Fungal protective cell wall, composed mainly of glucans, chitin and glycoproteins, whereas in Bacteria, the cell wall is composed of peptidoglycan. The antimicrobial effect observed by exposure to the extracts could possibly be attributed to a combination of these phytochemicals.

Antimicrobial activity of phenolic compounds has been previously reported with successful results *vs.* *E. coli* and *L. innocua*, however, doses up to 6 mg/mL were needed [32]. Additionally, there are some reports that state that specific phenolic compounds, such as anthocyanins, can affect mostly Gram-positive bacteria, by disrupting their cell wall and cytoplasmic membrane [33]. Our results provide valuable additional information, demonstrating that Gram-positive bacteria were more susceptible to the extract exposure; however, *R. discolor* extracts were also effective against Gram-negative *E. coli* [34]. There are several possible mechanisms by which phytochemicals can produce an antimicrobial effect: possible alteration of the physicochemical properties of the plasma membrane, pore formation, DNA gyrase and nucleic acid synthesis inhibition and toxicity through generation of hydrogen peroxide [35]. However, these mechanisms are not fully understood. The effect observed by the extracts on the tested microorganisms suggests that they could interfere directly with membrane structure by creating pores, due to the time required to produce inhibition. This mechanism could very well explain the obtained flow cytometry imaging, where cell membrane permeability is needed for propidium iodide (PI) to enter the cell and attach itself to the DNA, signaling the microorganisms as compromised [36]. Flow cytometry provided a key technique since it explicitly demonstrated a significant, in some cases almost total, growth inhibition in the bacterial and fungal populations as a response to the extracts.

Although extract-dose response was microorganism dependent, a defined dose dependent antimicrobial activity trend was not apparent. While we are unable to fully explain this lack of dose dependency, it is necessary to remember that these are crude extracts and the specific active ingredients of each extract, and at each dosage, have yet to be identified. We assume that the beneficial effects are probably not produced by a single molecule, but as a synergistic outcome of several types of phytochemicals, which could effectively increase or decrease a response at different dosages. Our observations lead us to conclude that there were optimal doses of different extracts that achieved maximum growth inhibition.

Various applications of these extracts, or their constituents, as antibacterial and antimycotics could be developed. Several cleaning products, mouth washes, soaps and shampoos and personal hygiene products contain a variety of plant extracts [37] such as: *Rosmarinus officinalis* [38], *Calendula officinalis* [39] and *Chamomilla recutita* [40]. On the other hand, while modern medicine is advancing at a very rapid rate, some of the side effects of conventional drugs can be problematic to patients. Hence, to overcome this problem, and to increase patient acceptance, a wide array of herbal products and plant extracts have been tried and tested [17]. Results obtained in this research demonstrate that extracts, such as those we obtained from *R. discolor*, could be applied as antiseptic agents for food and medical purposes. It is important to note that the most efficient extracts are water and polar solvent based. This represents a great advantage since pharmaceutical companies favor these procedures in the formulation and application of new products, due to their low health risk.

## 3. Experimental Section

### 3.1. Plant Material

*Rhoeo discolor* plants, also known as *Tradescantia spathacea*, and commonly referred to as “*Maguey morado*” or purple maguey, were collected from the town of Antón Lizardo (latitude: 19.0563842, longitude: −95.9878420) in the state of Veracruz, Mexico (botanical reference voucher No. CIB 14425). Leaves were washed with distilled water, excess water was blot-dried with paper towels and leaves were cut into 1 cm by 3 cm strips. Leaves were dried in a pilot plant Shel Lab drying oven at 40 °C for 24 h, with the exception of a small sample designated for an aqueous extract. Dry leaves had a mean loss of 94.3% of their weight in water after drying.

### 3.2. Aqueous Extract Preparations

Three aqueous extracts were obtained: (a) water dry leaf non-boiled: 5 g of dry leaves were submerged in 500 mL Mili-Q water and left to stir over-night; (b) water non-dried leaves non-boiled: 62.5 g of non-dried leaves were also left to stir overnight in 500 mL of Mili-Q water; and (c) water dry leaves boiled: 5 g of dry leaves were boiled for 30 min in 500 mL of Mili-Q water [1]. Excessive organic solid material was mechanically separated with a metal strainer to later proceed to filtration through a 0.22 μm membrane (Corning vacuum filtering system, Corning Incorporated, New York, NY, USA). Subsequently, extracts were frozen and then freeze-dried (Labconco FreeZone 12, Labconco Corporation, Kansas City, MO, USA) and stored at −20 °C until used.

### 3.3. Organic Solvent Extract Preparation

Several organic solvents with different polarities were used to obtain extracts, ensuring the extraction of a larger variety of molecules found in the plant. Standardized protocols for extracts required dried leaves [8]. Seven glass containers were filled with 900 mL of the selected solvents: methanol (MeOH), ethanol (EtOH), Ethyl Acetate (EtAc), Acetone (Ac), Petroleum Ether (PtEt), Chloroform (CHCl_3_) and Hexane (Hx), all supplied by Sigma Aldrich (St. Louis, MO, USA); 85 g of organic matter were introduced into each container [8]. The solvents and dried leaves were incubated at room temperature with constant stirring for 5 days. After incubation time, extracts were filtered twice with a vacuum filtering system using a Whatman No. 1 filter paper. This procedure was repeated twice. The extract was processed with a Heidolph Hei-Vap Precision Rotary Evaporator (Heidolph Instruments GmbH & Co.KG, Schwabach, Germany) to reduce volume and concentrate the extracts. Total dryness was obtained by exposing the remaining volumes to N_2_ flush. Resulting solids were then stored at −20 °C.

### 3.4. Dose Preparation

Stock doses were prepared at a concentration of 500 μg/mL, by dissolving 5 mg of dried extract in 10 mL of each corresponding solvent. Stock solutions were diluted to obtain the test concentration: 1, 1.5, 2, 2.5, 3, 3.5, 4 and 500 μg/mL; for a total of 80 doses. All doses were filtered with a 0.20 μm pore Corning sterile syringe filter in a sterile chamber and then stored at −20 °C.

### 3.5. Organisms and Growth Conditions

The following strains were used in this study: *Escherichia coli* ATCC 11229, *Pseudomonas aeruginosa* ATCC 27853, *Listeria innocua* ATCC 51742, *Streptococcus mutans* ATCC 31341, and lastly, the yeast *Candida albicans* ATCC 10231. Strains were stored in non-specific Trypticasein Soy Broth (TSB) (BD Bioxon, Estado de Mexico, Mexico) with 20% glycerol at −82 °C.

### 3.6. Inoculum Preparation

Bacterial and fungal inoculates were prepared from overnight culture suspensions in 50 mL of TSB. *E. coli*, *L. innocua* and *P. aeruginosa* were incubated at 37 °C for 48 h; *S. mutans* at 37 °C for 24 h; and *C. albicans* at 25 °C for 48 h. All cultures were incubated under aerobic conditions until exponential growth was reached [41]. Different temperatures and time periods were standardized to insure optimal culture growth conditions.

### 3.7. Agar-Disc Diffusion Assay

Whatman No. 1 filter paper circles were cut into 7 mm diameter discs; and were then placed in a glass petri dish for autoclave sterilization. *E. coli* was inoculated on to nutrient agar (Merck Chemicals, Darmstadt, Germany) plates; *L. innocua* on to Oxford Medium (BD Difco, Pont de Claix, France) plates; *Streptococcus mutans* on Brain-Heart-Infusion medium (BD Bioxon) plates; *Pseudomonas aeruginosa* on to Tryptic Soy Agar (TSA) (Merck Chemicals) plates; *Candida albicans* on to Potato Dextrose Agar (PDA) (BD Difco). For each dose, including stock solutions, three discs were placed on each plate with 13 μL of every test sample. Bacterial plates were incubated at 37 °C for 48 h, except for *S. mutans*, which was incubated at 37 °C for 24 h, and *C. albicans*, which was incubated at 25 °C for 24 h, to simulate the conditions required for their optimal growth in broth. The procedure was run in quadruplicate. Inhibition halos were measured and analyzed.

### 3.8. Total Phenolic Count

Total phenolic compounds were determined by a Folin-Ciocalteu’s assay. Samples were prepared at a concentration of 8.92 mg/mL. Briefly, in a 96-well plate, 240 µL of distilled water and 15 µL of the sample or blank solutions were added, followed by 15 µL of Folin-Ciocalteu’s phenol reagent (Sigma Aldrich). The plate was left to incubate in the dark for 3 min. Lastly, 30 µL of sodium carbonate (Na_2_CO_3_) were added and incubated for 2 h in the dark. Absorbance was measured at 725 nm. Total phenolic count was determined by using a gallic acid standard curve. Data was expressed as µg of gallic acid equivalent per 100 mg of sample (µg GAE/100 mg) and is presented in Table 2.

### 3.9. High-Performance Liquid Chromatography (HPLC) Analysis

All extracts were analyzed by reverse phase HPLC using an Agilent Technologies 1200 Series HPLC (Santa Clara, CA, USA) equipped with UV-visible and ELSD detectors. Chromatographic separation was performed using a Zorbax SB-Aq C18 column Solvent Saver Plus 3.0 × 150 mm, 3.5 µm (Santa Clara, CA, USA) [35]. Extracts were analyzed by three different elution methods, depending on the polarity of the solvents employed. Water, methanol and ethanol based extracts were analyzed by applying a gradient elution based on the variation of the proportion between solvent B (acetonitrile) to A (water). Separation was achieved by proportion variation: initially 15% of solvent B, increasing to 40% in 8 min, and then 100% solvent B in 10 min for a duration of 5 min [35,42,43]. Anthocyanin standards (cyanidin-3-glucoside and pelargonidin-3-glucoside) were obtained commercially from Chromadex (Laguna Hills, CA, USA) [42].

### 3.10. High-Performance Liquid Chromatograpjy Coupled with Mass Spectrometry-Time of flight (HPLC-MS-TOF) Analysis

Identification of anthocyanins was carried out by HPLC-MS-TOF (Agilent 1100). The same conditions from the HPLC analysis were applied to the HPLC-MS-TOF protocol. An electrospray source in positive mode (ESI^+^) was used to obtain the mass spectra under the following conditions: *m*/*z* range, 180–1500; nitrogen gas; gas temperature, 350 °C; drying gas flow rate, 13 L/min; nebulizer pressure, 50 psig; capillary voltage, 3000 V; and fragment voltage, 70 V. Anthocyanin mass was obtained using the Analyst QS 1.1 software (Applied Biosystems, Carlsbad, CA, USA) and compared with previous reports.

### 3.11. Flow Cytometry Analysis

In order to determine the bactericidal, bacteriostatic or fungicidal effect of the most significant extract-dose interactions, viability assays using PI (Clontech, Clontech Laboratories, Inc. A Takara Bio Company, Terra Bella Avenue, Mountain View, CA, USA) were performed. Briefly, microorganisms were grown in a TSB suspension; 1 mL of each culture was centrifuged at 13,400 rpm and re-suspended in PBS pH 7.4. Extracts were then added and incubated at 37 °C for bacteria and 25 °C for *C. albicans*, for 24 h. Vials were centrifuged again and bacterial and fungal pellets were re-suspended in PBS plus 5 µL of PI. Samples were incubated for 10 min in the dark at room temperature before processing. Samples were analyzed in a BD FACSCanto Flow Cytometer (BD Biosciences, Mississauga, ON, Canada), using a single laser emitting excitation light at 488 nm [44].

### 3.12. Statistical Analyses

Experiments were performed in quadruplicate with inner triplicates. Determinations of statistical significance were obtained by means of a one-way ANOVA conducted on Minitab v.17 (State College, PA, USA). Differences were considered significant at *p* < 0.05.

## 4. Conclusions

The wide inhibition effect noted when microorganisms were exposed to the different extracts demonstrated some tendencies towards better functionally of the polar extracts. Nevertheless, in some cases, both polar and non-polar extracts resulted in a significant inhibitory effect. It can only be hypothesized that these beneficial effects are probably not produced by a single molecule, but as a synergistic outcome of several types of phytochemicals. With further analysis it could be assumed that these natural extracts have potential uses as food and cosmetic preservatives, functional food additives and even for medical applications. However, further research is needed in order to determine the effective extract amounts to inhibit microbial pathogen growth in food systems and cytotoxicity.

## Supplementary Materials

Supplementary materials can be accessed at: http://www.mdpi.com/1420-3049/20/10/18685/s1.

## References

[1] Rosales-Reyes T., de la Garza M., Arias-Castro C., Rodriguez-Mendiola M., Fattel-Fazenda S., Arce-Popoca E., Hernandez-Garcia S., Villa-Trevino S. (2008). Aqueous crude extract of *Rhoeo discolor*, a mexican medicinal plant, decreases the formation of liver preneoplastic foci in rats. J. Ethnopharmacol..

[2] Gonzalez-Avila M., Arriaga-Alba M., de la Garza M., del Carmen H.M., Dominguez-Ortiz M.A., Fattel-Fazenda S., Villa-Trevino S. (2003). Antigenotoxic, antimutagenic and ros scavenging activities of a *Rhoeo discolor* ethanolic crude extract. Toxicol. In Vitro.

[3] Giovannini P., Reyes-Garcia V., Waldstein A., Heinrich M. (2011). Do pharmaceuticals displace local knowledge and use of medicinal plants? Estimates from a cross-sectional study in a rural indigenous community, mexico. Soc. Sci. Med..

[4] Mosa A.I., Emara A.A., Yousef J.M., Saddiq A.A. (2011). Novel transition metal complexes of 4-hydroxy-coumarin-3-thiocarbohydrazone: Pharmacodynamic of Co(Iii) on rats and antimicrobial activity. Spectrochim. Acta A Mol. Biomol. Spectrosc..

[5] Wattenberg L.W. (1992). Inhibition of carcinogenesis by minor dietary constituents. Cancer Res..

[6] Martin C., Zhang Y., Tonelli C., Petroni K. (2013). Plants, diet, and health. Annu. Rev. Plant Biol..

[7] Idaka E., Ogawa T., Kondo T., Goto T. (1987). Isolation of highly acylated anthocyanins from commelinaceae plants, zebrina pendula, rhoeo spathacea and setcreasea purpurea. Agric. Biol. Chem..

[8] Arriaga-Alba M., Blasco J.L., Ruiz-Perez N.J., Sanchez-Navarrete J., Rivera-Sanchez R., Gonzalez-Avila M. (2011). Antimutagenicity mechanisms of the *Rhoeo discolor* ethanolic extract. Exp. Toxicol. Pathol..

[9] Arenas-Hernandez M.M., Martinez-Laguna Y., Torres A.G. (2012). Clinical implications of enteroadherent *Escherichia coli*. Curr. Gastroenterol. Rep..

[10] Hoiby N., Frederiksen B., Pressler T. (2005). Eradication of early *Pseudomonas aeruginosa* infection. J. Cyst. Fibros..

[11] Siegman-Igra Y., Levin R., Weinberger M., Golan Y., Schwartz D., Samra Z., Konigsberger H., Yinnon A., Rahav G., Keller N. (2002). Listeria monocytogenes infection in israel and review of cases worldwide. Emerg. Infect. Dis..

[12] Wilkinson P.J. (1989). Ignorance about listeria. BMJ.

[13] Forssten S.D., Bjorklund M., Ouwehand A.C. (2010). *Streptococcus mutans*, caries and simulation models. Nutrients.

[14] Moyes D.L., Naglik J.R. (2011). Mucosal immunity and *Candida albicans* infection. Clin. Dev. Immunol..

[15] Tan J.B.L., Yap W.J., Tan S.Y., Lim Y.Y., Lee S.M. (2014). Antioxidant content, antioxidant activity, and antibacterial activity of five plants from the commelinaceae family. Antioxidants.

[16] Tan J.B.L., Lim Y.Y., Lee S.M. (2013). Antioxidant and antibacterial activity of rhoeo spathacea (swartz) stearn leaves. J. Food Sci. Technol..

[17] Praveen N.C., Rajesh A., Madan M., Chaurasia V.R., Hiremath N.V., Sharma A.M. (2014). *In vitro* evaluation of antibacterial efficacy of pineapple extract (bromelain) on periodontal pathogens. J. Int. Oral Health.

[18] Garcia-Garcia R., Lopez-Malo A., Palou E. (2011). Bactericidal action of binary and ternary mixtures of carvacrol, thymol, and eugenol against *Listeria innocua*. J. Food Sci..

[19] Garcia-Garcia R., Escobedo-Avellaneda Z., Tejada-Ortigoza V., Martin-Belloso O., Valdez-Fragoso A., Welti-Chanes J. (2015). Hurdle technology applied to prickly pear beverages for inhibiting saccharomyces cerevisiae and *Escherichia coli*. Lett. Appl. Microbiol..

[20] Pedras M.S., Yaya E.E. (2015). Plant chemical defenses: Are all constitutive antimicrobial metabolites phytoanticipins?. Nat. Prod. Commun..

[21] Cushnie T.P., Lamb A.J. (2005). Antimicrobial activity of flavonoids. Int. J. Antimicrob. Agents.

[22] Mujeeb F., Bajpai P., Pathak N. (2014). Phytochemical evaluation, antimicrobial activity, and determination of bioactive components from leaves of aegle marmelos. Biomed. Res. Int..

[23] Tatsuzawa F., Saito N., Maeyama K., Yokoi M., Shigihara A., Honda T. (2010). Triacylated anthocyanidin 3-arabinosylglucoside-7,3ʹ-diglucosides isolated from the bluish flowers of tradescantia virginiana cultivars and their distribution in the tradescantieae.

[24] Cisowska A., Wojnicz D., Hendrich A.B. (2011). Anthocyanins as antimicrobial agents of natural plant origin. Nat. Prod. Commun..

[25] Mira N.V.M., Barros R.M.C., Schiocchet M.A., Noldin J.A., Lanfer-Marquez U.M. (2008). Extraction, analysis and distribution of phenolic acids in pigmented and non-pigmented genotypes of rice (*Oryza sativa* L.). Ciênc. Tecnol. Aliment..

[26] Gardjeva P.A., Dimitrova S.Z., Kostadinov I.D., Murdjeva M.A., Peyche L.P., Lukanov L.K., Stanimirova I.V., Alexandrov A.S. (2007). A study of chemical composition and antimicrobial activity of bulgarian propolis. Folia Med.(Plovdiv.).

[27] Mueller U., Sauer T., Weigel I., Pichner R., Pischetsrieder M. (2011). Identification of H_2_O_2_ as a major antimicrobial component in coffee. Food Funct..

[28] Kozyra M., Biernasiuk A., Malm A., Chowaniec M. (2015). Chemical compositions and antibacterial activity of extracts obtained from the inflorescences of *Cirsium canum* (L.) all. Nat. Prod. Res..

[29] Popova M.P., Chinou I.B., Marekov I.N., Bankova V.S. (2009). Terpenes with antimicrobial activity from cretan propolis. Phytochemistry.

[30] Wang J., Cao F., Su E., Wu C., Zhao L., Ying R. (2013). Improving flavonoid extraction from ginkgo biloba leaves by prefermentation processing. J. Agric. Food Chem..

[31] Harborne J.B., Williams C.A. (2000). Advances in flavonoid research since 1992. Phytochemistry.

[32] Tekeli Y., Karpuz E., Danahaliloglu H., Bucak S., Guzel Y., Erdmann H. (2014). Phenolic composition, antioxidant capacity of salvia verticcilata and effect on multidrug resistant bacteria by flow-cytometry. Afr. J. Tradit. Complement Altern. Med..

[33] Patra J.K., Kim E.S., Oh K., Kim H.J., Kim Y., Baek K.H. (2014). Antibacterial effect of crude extract and metabolites of phytolacca americana on pathogens responsible for periodontal inflammatory diseases and dental caries. BMC. Complement Altern. Med..

[34] Tan J.B., Lim Y.Y. (2015). Critical analysis of current methods for assessing the *in vitro* antioxidant and antibacterial activity of plant extracts. Food Chem..

[35] De Araujo A.A., Soares L.A., Assuncao Ferreira M.R., de Souza Neto M.A., da Silva G.R., de Araujo R.F.J., Guerra G.C., de Melo M.C. (2014). Quantification of polyphenols and evaluation of antimicrobial, analgesic and anti-inflammatory activities of aqueous and acetone-water extracts of libidibia ferrea, parapiptadenia rigida and psidium guajava. J. Ethnopharmacol..

[36] Tamburini S., Ballarini A., Ferrentino G., Moro A., Foladori P., Spilimbergo S., Jousson O. (2013). Comparison of quantitative pcr and flow cytometry as cellular viability methods to study bacterial membrane permeabilization following supercritical CO_2_ treatment. Microbiology.

[37] Herman A., Herman A.P., Domagalska B.W., Mlynarczyk A. (2013). Essential oils and herbal extracts as antimicrobial agents in cosmetic emulsion. Indian J. Microbiol..

[38] Hofling J.F., Anibal P.C., Obando-Pereda G.A., Peixoto I.A., Furletti V.F., Foglio M.A., Goncalves R.B. (2010). Antimicrobial potential of some plant extracts against candida species. Braz. J. Biol..

[39] Efstratiou E., Hussain A.I., Nigam P.S., Moore J.E., Ayub M.A., Rao J.R. (2012). Antimicrobial activity of *Calendula officinalis* petal extracts against fungi, as well as gram-negative and gram-positive clinical pathogens. Complement Ther. Clin. Pract..

[40] Agusti G., Fittipaldi M., Morato J., Codony F. (2013). Viable quantitative pcr for assessing the response of *Candida albicans* to antifungal treatment. Appl. Microbiol. Biotechnol..

[41] Assaf A.M., Haddadin R.N., Aldouri N.A., Alabbassi R., Mashallah S., Mohammad M., Bustanji Y. (2013). Anti-cancer, anti-inflammatory and anti-microbial activities of plant extracts used against hematological tumors in traditional medicine of jordan. J. Ethnopharmacol..

[42] Urias-Lugo D.A., Heredia J.B., Muy-Rangel M.D., Valdez-Torres J.B., Serna-SaldÃ­var S.O., GutiÃ©rrez-Uribe J.A. (2015). Anthocyanins and phenolic acids of hybrid and native blue maize (*Zea mays* L.) extracts and their antiproliferative activity in mammary (MCF7), liver (HepG2), colon (Caco2 and HT29) and prostate (PC3) cancer cells. Plant Foods Hum. Nutr..

[43] Zare K., Movafeghi A., Mohammadi S.A., Asnaashari S., Nazemiyeh H. (2014). New phenolics from *Linum mucronatum* subsp. Orientale. Bioimpacts..

[44] Berney M., Hammes F., Bosshard F., Weilenmann H.U., Egli T. (2007). Assessment and interpretation of bacterial viability by using the live/dead baclight kit in combination with flow cytometry. Appl. Environ. Microbiol..

