# Supplementary material for: Antimicrobial Activity of Rhoeo discolor Phenolic Rich Extracts Determined by Flow Cytometry
Source: Molecules. 2015 Oct 14;20(10):18685–703. doi: 10.3390/molecules201018685 (PMC6331916; doi:10.3390/molecules201018685)
Supplement: Supplementary file 1 [file molecules-20-18685-s001.pdf]

Supplementary Materials

Inhibition halos in Agar-disc diffusion assay

No inhibition halos | **Inhibition halos present**

E. coli

Chloroform extract, doses 3 µg/mL (A) and 3.5 µg/mL (B). This plate shows no inhibition halos

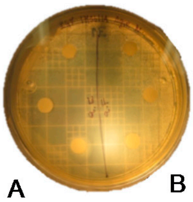

A B

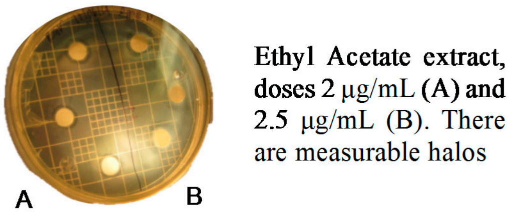

A B

Ethyl Acetate extract, doses 2 µg/mL **(A) and** 2.5 µg/mL (B). There are measurable halos

L. innocua

Water dry leaf boiled (A) and non-boiled extracts (B), doses 500 µg/mL. No inhibition was observed

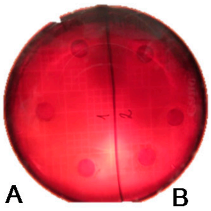

A B

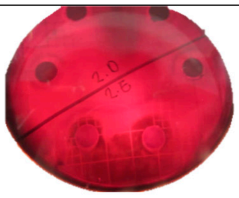

A B

Water dry leaf non-boiled extract, doses 2.5 µg/mL (A) and 3 µg/mL (B). Important inhibition can be observed

S. mutans

Chloroform extract, doses 2 µg/mL (A) and 2.5 µg/mL (B). No inhibition was observed

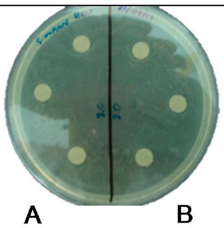

A B

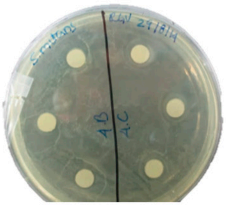

A B

Methanol extract, doses 2.5 µg/mL (A) **and** 3 µg/mL (B). Inhibition can be observed

C. albicans

Acetone extract, doses 1.5 µg/mL (A) and 2 µg/mL (B). No significant inhibition was observed

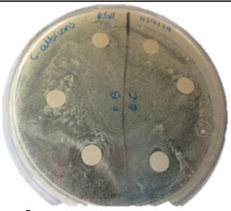

A B

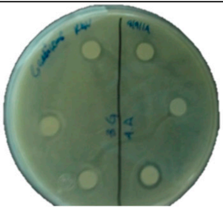

A B

Water non-dried leafs non-boiled extract, dose 4 µg/mL **(A) and Methanol extract dose** 1 µg/mL **(B)**

P. aeruginosa

Water dry leaf boiled extract, doses 1 µg/mL (A) and 1.5 µg/mL (B). No inhibition was observed

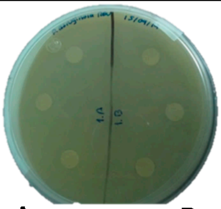

A B

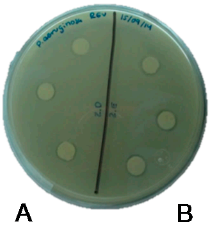

A B

Water non-dried leaves non-boiled extract, dose 4 µg/mL (A) **and Methanol extract dose** 1 µg/mL (B). No significant inhibition

Figure S1. Extract agar-disc diffusion assay. The extracts were placed on filter paper discs, which diffuse the extract into the medium. When an extract is bioactive, an inhibition halo forms around the disc, preventing microorganism growth.

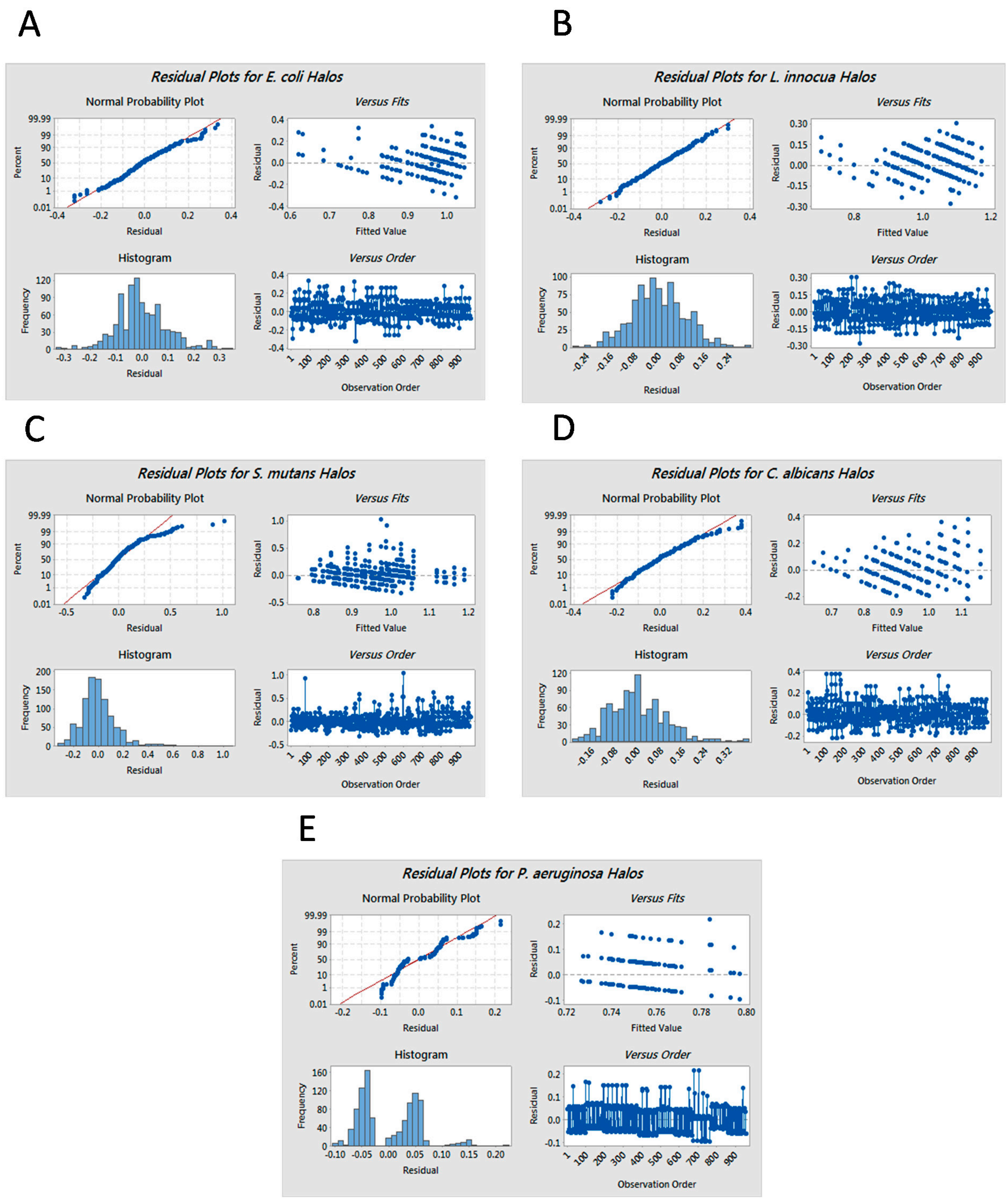

Figure S2. Halo residual plots (**A**) *E. coli*; (**B**) *L. innocua*; (**C**) *S. mutans*; (**D**) *C. albicans*; and (**E**) *P. aeruginosa*. These charts provide information on the uniformity of the results, indicating repeatability and leaving little error among samples.

Table S1. Inhibition halos measurements extract-dose interaction.

Dose	*E. coli*	*L. innocua*	*S. mutans*	*C. albicans*	*P. aeruginosa*
**Extract #1 Water, Dry Leaf, Non-Boiled**					
500 µg/mL	0.70	0.94	0.96	0.89	0.75
1 µg/mL	0.94	1.08	* 1.22	* 1.19	0.77
1.5 µg/mL	0.85	1.07	* 1.14	0.93	0.76
2 µg/mL	0.94	* 1.17	* 1.11	1.01	0.78
2.5 µg/mL	0.91	* 1.13	* 1.18	1.04	0.74
3 µg/mL	0.97	* 1.11	1.06	1.03	0.76
3.5 µg/mL	* 1.13	* 1.16	1.09	0.98	0.74
4 µg/mL	* 1.13	* 1.23	1.06	* 1.14	0.70
**Extract #2 Water, Non-Dried Leaf, Non-Boiled**					
500 µg/mL	0.70	0.98	0.88	0.92	0.78
1 µg/mL	0.97	* 1.15	1.04	* 1.19	0.70
1.5 µg/mL	* 1.18	1.08	* 1.11	* 1.40	0.70
2 µg/mL	1.01	* 1.18	1.01	* 1.18	0.70
2.5 µg/mL	0.99	* 1.21	0.79	0.98	0.77
3 µg/mL	0.90	* 1.11	0.85	0.99	0.77
3.5 µg/mL	0.81	1.03	0.95	1.03	0.73
4 µg/mL	1.08	0.91	0.98	1.06	0.73
**Extract #3 Water, Dry Leaf, Boiled**					
500 µg/mL	0.70	0.70	0.95	0.75	0.87
1 µg/mL	0.98	0.94	0.97	0.88	0.73
1.5 µg/mL	0.98	1.03	1.05	0.98	0.73
2 µg/mL	1.03	* 1.23	0.89	0.77	0.73
2.5 µg/mL	1.03	* 1.12	0.88	0.83	0.73
3 µg/mL	0.91	1.08	0.88	1.01	0.70
3.5 µg/mL	0.91	1.05	0.98	0.76	0.70
4 µg/mL	0.91	* 1.11	* 1.14	1.03	0.80
**Extract #4 Methanol**					
500 µg/mL	0.70	0.85	0.87	0.75	0.82
1 µg/mL	0.94	* 1.17	* 1.19	* 1.20	0.81
1.5 µg/mL	0.96	0.98	0.98	0.95	0.73
2 µg/mL	1.02	1.03	0.95	0.94	0.73
2.5 µg/mL	1.02	* 1.16	0.86	1.01	0.73
3 µg/mL	1.09	1.09	0.91	0.92	0.73
3.5 µg/mL	1.02	1.08	0.85	1.04	0.73
4 µg/mL	1.07	1.08	0.90	0.93	0.73
**Extract #5 Ethanol**					
500 µg/mL	0.70	0.73	0.85	0.73	0.75
1 µg/mL	0.97	* 1.14	0.81	0.98	0.73
1.5 µg/mL	1.07	* 1.17	1.02	0.97	0.73
2 µg/mL	1.00	0.98	0.88	0.93	0.79

Table S1. *Cont.*

Dose	*E. coli*	*L. innocua*	*S. mutans*	*C. albicans*	*P. aeruginosa*
**Extract #5 Ethanol**					
2.5 µg/mL	0.97	1.03	1.03	0.94	0.77
3 µg/mL	1.02	* 1.10	0.98	0.88	0.82
3.5 µg/mL	1.02	1.07	0.93	0.90	0.82
4 µg/mL	* 1.23	* 1.14	0.83	0.88	0.72
**Extract #6 Ethyl Acetate**					
500 µg/mL	0.70	0.72	0.81	0.73	0.75
1 µg/mL	* 1.15	1.05	0.97	0.83	0.72
1.5 µg/mL	1.02	1.04	0.89	0.75	0.72
2 µg/mL	0.75	* 1.13	0.97	0.75	0.72
2.5 µg/mL	1.02	1.03	0.90	1.03	0.73
3 µg/mL	0.90	1.08	0.83	0.83	0.73
3.5 µg/mL	0.89	0.98	0.95	0.89	0.83
4 µg/mL	0.75	0.89	0.96	0.86	0.83
**Extract #7 Acetone**					
500 µg/mL	0.74	0.89	0.78	0.72	0.73
1 µg/mL	0.88	0.98	1.01	0.89	0.78
1.5 µg/mL	1.01	0.97	1.00	0.89	0.78
2 µg/mL	0.94	0.95	0.98	0.88	0.74
2.5 µg/mL	1.05	0.90	0.86	1.08	0.77
3 µg/mL	* 1.10	1.00	0.84	0.92	0.76
3.5 µg/mL	0.68	0.93	0.84	0.95	0.77
4 µg/mL	1.02	0.99	* 1.18	0.96	0.78
**Extract #8 Petroleum Ether**					
500 µg/mL	0.73	0.70	0.81	0.75	0.73
1 µg/mL	0.94	1.01	* 1.29	* 1.15	0.82
1.5 µg/mL	0.93	1.08	1.00	0.99	0.84
2 µg/mL	0.85	1.03	* 1.19	1.05	0.81
2.5 µg/mL	0.94	0.92	0.80	0.95	0.80
3 µg/mL	0.88	0.92	0.80	1.07	0.78
3.5 µg/mL	0.89	0.98	0.94	0.88	0.78
4 µg/mL	0.89	0.96	0.93	0.94	0.78
**Extract #9 Chloroform**					
500 µg/mL	0.75	0.78	0.70	0.70	0.70
1 µg/mL	0.79	1.00	0.85	0.79	0.74
1.5 µg/mL	0.97	0.98	0.84	0.83	0.73
2 µg/mL	0.83	1.05	0.70	0.74	0.70
2.5 µg/mL	0.89	0.95	0.70	0.78	0.70
3 µg/mL	0.79	0.83	1.03	0.88	0.76
3.5 µg/mL	0.78	0.83	* 1.19	0.88	0.77
4 µg/mL	0.77	0.93	0.97	0.83	0.80

Table S1. *Cont.*

Dose	*E. coli*	*L. innocua*	*S. mutans*	*C. albicans*	*P. aeruginosa*
**Extract #10 Hexane**					
500 µg/mL	0.72	0.83	0.70	0.70	0.70
1 µg/mL	0.84	0.98	0.97	0.81	0.80
1.5 µg/mL	0.84	0.91	1.02	0.83	0.76
2 µg/mL	0.83	0.93	* 1.11	0.84	0.78
2.5 µg/mL	0.84	0.84	0.72	0.88	0.76
3 µg/mL	0.97	0.91	0.70	0.99	0.77
3.5 µg/mL	0.81	0.90	0.88	0.96	0.75
4 µg/mL	0.81	0.86	0.88	0.83	0.75

Numerical data is represented in centimeters (cm) and corresponds to the halo diameter for each disc and extract sample. The diameter of the filter discs measured 0.7 cm, therefore measurements in excess of 7 cm will indicate an effect of the extract and dose on a particular microorganism. Diameters labeled with * correspond to the most significant growth inhibition effects. All standard deviations were found to be <10%, and $p < 0.05$.
